# Supplementary material for: A role for ETV1 and endothelial cell-derived extracellular vesicle microRNAs in priming fibroblast response to vesicle-bound FGF2
Source: bioRxiv. 2026 Feb 25:2026.02.25.707568. Preprint. [Version 1] doi: 10.64898/2026.02.25.707568 (PMC13160119; doi:10.64898/2026.02.25.707568)
Supplement: Supplement 1 [file NIHPP2026.02.25.707568v1-supplement-1.pdf]

bioRxiv preprint doi: https://doi.org/10.64898/2026.02.25.707568; this version posted February 25, 2026. The copyright holder for this preprint (which was not certified by peer review) is the author/funder, who has granted bioRxiv a license to display the preprint in perpetuity. It is made available under aCC-BY 4.0 International license.

Supplementary Tables

Table S1. siRNA and miRNA mimics used for transfection

siRNA or mimic	Assay ID	Catalog #	Source	Final concentration
Silencer™ Cy™3 Labeled Negative Control #1 siRNA		AM4621	Thermo Fisher Scientific	10 nM
ETV1 Silencer® siRNA	115583	AM16708	Thermo Fisher Scientific	10 nM
Negative Control #1 miRVana™ miRNA mimic		4464058	Thermo Fisher Scientific	20 nM
hsa-miR-126-3p mirVana® miRNA mimic	MC12841	4464066	Thermo Fisher Scientific	20 nM
hsa-miR-151a-3p mirVana® miRNA mimic	MC11405	4464066	Thermo Fisher Scientific	20 nM
hsa-miR-99a-3p mirVana® miRNA mimic	MC10719	4464066	Thermo Fisher Scientific	20 nM
hsa-miR-21-5p mirVana® miRNA mimic	MC10206	4464066	Thermo Fisher Scientific	20 nM
hsa-miR-221-3p mirVana® miRNA mimic	MC10337	4464066	Thermo Fisher Scientific	20 nM

Table S2. Primer sequences for fibroblast gene and miRNA expression analysis

Gene or miRNA	Forward (5’ → 3’)	Reverse (3’ → 5’)
GAPDH	CAGGGCTGCTTTTAACTCTGG	TGGGTGGAATCATATTGGAACA
ETV1	CCAGCTTTCTGAACCCTGTA	TGTTCATACACTGGGTCGTG
ACTA2	AATGCAGAAGGAGATCACGG	TCCTGTTTGCTGATCCACATC
COL1A2	AGGACAAGAAACACGTCTGG	GGTGATGTTCTGAGAGGCATAG
COL3A1	AAGTCAAGGAGAAAGTGGTCG	CTCGTTCTCCATTCTTACCAGG
FN1	CGGTGGCTGTCAGTCAAAG	AAACCTCGGCTTCCTCCATAA
ELN	GCAGGAGTTAAGCCCAAGG	TGTAGGGCAGTCCATAGCCA
MMP1	AAAATTACACGCCAGATTTGCC	GGTGTGACATTACTCCAGAGTTG
ITGA11	GTGGCAATAAGTGGCTGGTC	GTTCCCGTGGATCACTGGAC
hsa-RNU6B	GCCCCTGCGCAAGGATGAC	
hsa-miR-126-3p	GGTCGTACCGTGAGTAATAATGCG	
hsa-miR-21-5p	GGGTAGCTTATCAGACTGATGTTGA	
hsa-miR-99a-5p	GAACCCGTAGATCCGATCTTGTG	
hsa-miR-151a-3p	GCTAGACTGAAGCTCCTTGAGG	
hsa-miR-10b-5p	GGTACCCTGTAGAACCGAATTTGTG	
hsa-miR-320a-3p	AAAAGCTGGGTTGAGAGGGCGA	
hsa-miR-1246	GGGAATGGATTTTTGGAGCAGG	
hsa-miR-146a-5p	GGTGAGAACTGAATTCCATGGGTT	

bioRxiv preprint doi: https://doi.org/10.64898/2026.02.25.707568; this version posted February 25, 2026. The copyright holder for this preprint (which was not certified by peer review) is the author/funder, who has granted bioRxiv a license to display the preprint in perpetuity. It is made available under aCC-BY 4.0 International license.

Supplementary Figure Legends

Figure S1. Transfection of dermal fibroblasts with mimics of top 5 ECEV miRNAs fails to re-capitulate proliferative fibroblast phenotype induced by ECEVs. MTS assay was performed on dermal fibroblasts transfected with mimics of the top 5 indicated ECEV miRNAs to assess proliferation. OD values were normalized to the initial cell density. Data is presented as mean ± SD with data points indicating individual values from quadruplicate cultures. Two-way ANOVA with Bonferroni post-hoc test was used and no significant differences between groups were found. NCM, negative control mimic; OD, optical density.

Figure S2. ECEV treatment of fibroblasts leads to detectable transfer of miR-126-3p but not other top ECEV miRNAs. Fibroblasts treated with ECEVs for 24h were analyzed for miRNA expression analysis via RT-PCR. Relative expression is normalized to RNU6B. Data is presented as mean ± SD with data points indicating individual values from quadruplicate cultures. Two-way ANOVA with Bonferroni post-hoc test was used. ns = $p > 0.05$, * = $p < 0.05$, ** = $p < 0.01$, *** = $p < 0.001$, **** = $p < 0.0001$. ECEV, endothelial cell-derived extracellular vesicle; FB, fibroblast.

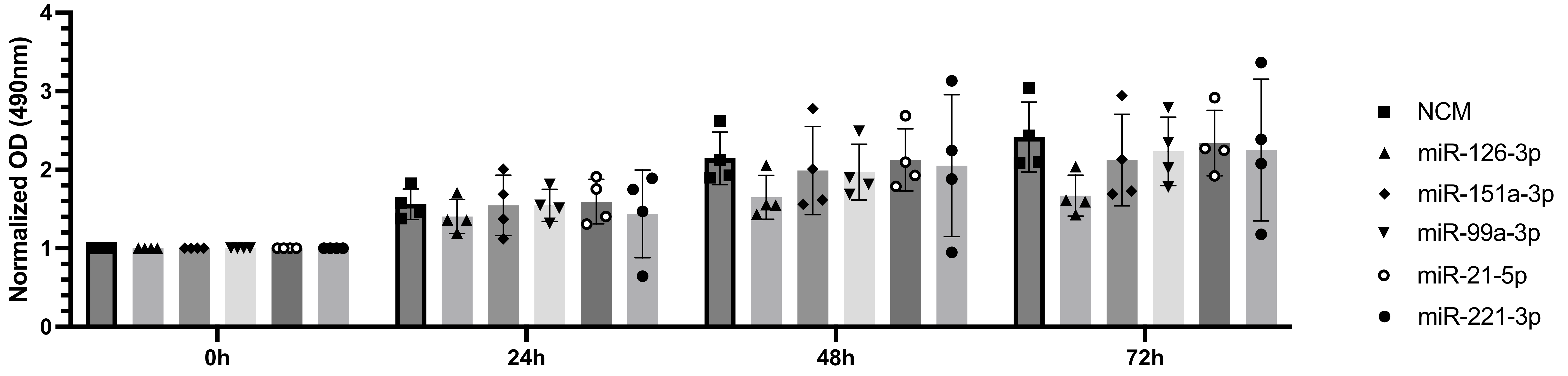

Figure S1. Transfection of dermal fibroblasts with mimics of top 5 ECEV miRNAs fails to re-capitulate proliferative fibroblast phenotype induced by ECEVs. MTS assay was performed on dermal fibroblasts transfected with mimics of the top 5 indicated ECEV miRNAs to assess proliferation. OD values were normalized to the initial cell density. Data is presented as mean ± SD with data points indicating individual values from quadruplicate cultures. Two-way ANOVA with Bonferroni post-hoc test was used and no significant differences between groups were found. NCM, negative control mimic; OD, optical density.

bioRxiv preprint doi: https://doi.org/10.64898/2026.02.25.707568; this version posted February 25, 2026. The copyright holder for this preprint (which was not certified by peer review) is the author/funder, who has granted bioRxiv a license to display the preprint in perpetuity. It is made available under aCC-BY 4.0 International license.

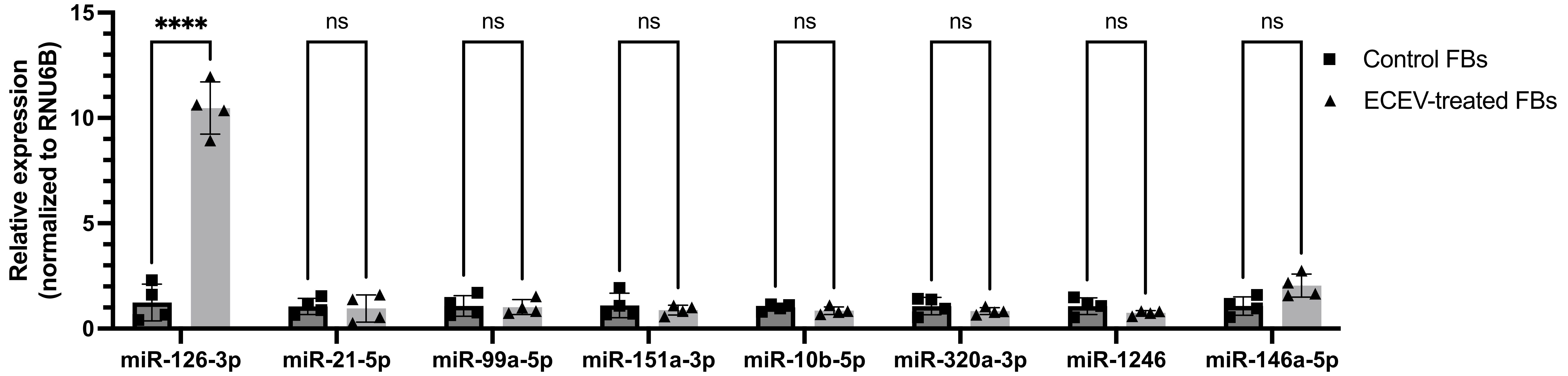

Figure S2. ECEV treatment of fibroblasts leads to detectable transfer of miR-126-3p but not other top ECEV miRNAs. Fibroblasts treated with ECEVs for 24h were analyzed for miRNA expression analysis via RT-PCR. Relative expression is normalized to RNU6B. Data is presented as mean ± SD with data points indicating individual values from quadruplicate cultures. Two-way ANOVA with Bonferroni post-hoc test was used. ns = p > 0.05, * = p < 0.05, ** = p < 0.01, *** = p < 0.001, **** = p < 0.0001. ECEV, endothelial cell-derived extracellular vesicle; FB, fibroblast.

bioRxiv preprint doi: https://doi.org/10.64898/2026.02.25.707568; this version posted February 25, 2026. The copyright holder for this preprint (which was not certified by peer review) is the author/funder, who has granted bioRxiv a license to display the preprint in perpetuity. It is made available under aCC-BY 4.0 International license.
